# Comparison of *In Vivo* and *Ex Vivo* MRI for the Detection of Structural Abnormalities in a Mouse Model of Tauopathy

**DOI:** 10.3389/fninf.2017.00020

**Published:** 2017-03-31

**Authors:** Holly E. Holmes, Nick M. Powell, Da Ma, Ozama Ismail, Ian F. Harrison, Jack A. Wells, Niall Colgan, James M. O'Callaghan, Ross A. Johnson, Tracey K. Murray, Zeshan Ahmed, Morten Heggenes, Alice Fisher, M. Jorge Cardoso, Marc Modat, Michael J. O'Neill, Emily C. Collins, Elizabeth M. C. Fisher, Sébastien Ourselin, Mark F. Lythgoe

**Affiliations:** ^1^Division of Medicine, UCL Centre for Advanced Biomedical Imaging, University College LondonLondon, UK; ^2^Centre for Medical Image Computing, University College LondonLondon, UK; ^3^Tailored Therapeutics, Eli Lilly and Company, Lilly Corporate CenterIndianapolis, IN, USA; ^4^Molecular Pathology, Eli Lilly & Co. LtdWindlesham, UK; ^5^Department of Neurodegenerative Disease, Institute of Neurology, University College LondonLondon, UK

**Keywords:** MRI imaging, preclinical imaging, *in vivo* imaging, phenotyping, tensor-based morphometry, tauopathy, mouse models, neurodegeneration

## Abstract

With increasingly large numbers of mouse models of human disease dedicated to MRI studies, compromises between *in vivo* and *ex vivo* MRI must be fully understood in order to inform the choice of imaging methodology. We investigate the application of high resolution *in vivo* and *ex vivo* MRI, in combination with tensor-based morphometry (TBM), to uncover morphological differences in the rTg4510 mouse model of tauopathy. The rTg4510 mouse also offers a novel paradigm by which the overexpression of mutant tau can be regulated by the administration of doxycycline, providing us with a platform on which to investigate more subtle alterations in morphology with morphometry. Both *in vivo* and *ex vivo* MRI allowed the detection of widespread bilateral patterns of atrophy in the rTg4510 mouse brain relative to wild-type controls. Regions of volume loss aligned with neuronal loss and pathological tau accumulation demonstrated by immunohistochemistry. When we sought to investigate more subtle structural alterations in the rTg4510 mice relative to a subset of doxycycline-treated rTg4510 mice, *ex vivo* imaging enabled the detection of more regions of morphological brain changes. The disadvantages of *ex vivo* MRI may however mitigate this increase in sensitivity: we observed a 10% global shrinkage in brain volume of the post-mortem tissues due to formalin fixation, which was most notable in the cerebellum and olfactory bulbs. However, many central brain regions were not adversely affected by the fixation protocol, perhaps due to our “in-skull” preparation. The disparity between our TBM findings from *in vivo* and *ex vivo* MRI underlines the importance of appropriate study design, given the trade-off between these two imaging approaches. We support the utility of *in vivo* MRI for morphological phenotyping of mouse models of disease; however, for subtler phenotypes, *ex vivo* offers enhanced sensitivity to discrete morphological changes.

## Introduction

Since Nature published the initial sequence of the (Mouse Genome Sequencing Consortium et al., 2002), there has been a marked increase in the number of transgenic and gene-targeted mice that have been engineered to deepen our understanding of the function of genes in human biology. It has been estimated that to create a knock-out mouse for each of the 20,000 genes in the mouse genome, over 7 million animals will be required in order to fully characterize the subsequent loss of gene activity (Qiu, 2006). This figure does not include knock-in and transgenic mice, all of which will also require characterization in order to fully comprehend gene function.

In order to analyse complex phenotypes, robust and reproducible methods for phenotyping are required (Gates et al., 2011). Biochemical, behavioral, anatomical, physiological and pathological assays all contribute to understanding the true function of a gene (Crawley, 2008). Phenotyping, at the macroscopic and microscopic level, is traditionally carried out using histological methods, which are useful for validating hypotheses and uncovering unexpected biochemical changes that accompany altered gene function. However, preparation of the tissues for histological analysis is terminal, invasive and limited by low throughput. Recently, techniques for structural phenotyping have moved beyond the use of histology to embrace whole-organ or organism, high resolution imaging methods (Turnbull and Mori, 2007; Lau et al., 2008; Carroll et al., 2011; Cleary et al., 2011a,b; Lerch et al., 2011a; Yu et al., 2011; Badhwar et al., 2013; Ellegood et al., 2013; Norris et al.).

Magnetic resonance imaging (MRI) is a powerful, non-invasive imaging technology which is increasingly employed for phenotyping transgenic mice; this is largely due to advances in MRI hardware and computer power, which facilitate the application of sophisticated image processing techniques (Benveniste and Blackband, 2002; Ma et al., 2005, 2008, 2014; McConville et al., 2005; Lau et al., 2008; Lerch et al., 2008a,b, 2011b; Spring et al., 2010; Zhang et al., 2010; Badhwar et al., 2013; Hébert et al., 2013). Through a combination of high resolution MRI and automated computational analysis, the throughput and efficiency of structural phenotyping of transgenic mice will significantly improve.

One of the key experimental decisions when scanning a mouse brain for phenotypic changes is whether to image the animal alive or dead in order to maximize sensitivity to morphological alterations with respect to wild-type (WT) controls (Lerch et al., 2012). Post-mortem imaging enables longer scan times, the use of contrast agents in high concentrations and limits motion. These factors can boost resolution, signal-to-noise ratio (SNR) and contrast-to-noise ratio (CNR) to support advanced, unbiased computational approaches such as tensor-based morphometry (TBM) (Lerch et al., 2008b; Ma et al., 2014). In addition, multiple brains can be imaged simultaneously using standard MRI hardware, thereby improving the throughput of data acquisition (Bock et al., 2005). Conversely, live animals' tissues do not suffer distortions and dehydration from fixation, which may disrupt their integrity (Cahill et al., 2012). Crucially, *in vivo* imaging permits longitudinal studies, where the same animal can be serially assessed to observe morphological and functional changes over time (Lau et al., 2008; Zhang et al., 2010). This has been shown to improve the statistical power of results (Lerch et al., 2012) as well as reduce the number of animals required for imaging studies, compared to cross-sectional studies.

In this study, we sought to expand upon our previous findings (Lerch et al., 2012), by investigating the sensitivity of *in vivo* and *ex vivo* MRI to morphological differences occurring in the rTg4510 mouse model of tauopathy (SantaCruz et al., 2005). The rTg4510 mouse exhibits gross atrophy of the forebrain regions, and has been extensively characterized in previous *in vivo* MRI studies (Yang et al., 2011; Wells et al., 2015; Holmes et al., 2016). Furthermore, the rTg4510 mouse offers a novel paradigm to support our investigation, as the overexpression of tau can be inactivated by the administration of doxycycline (SantaCruz et al., 2005). Therefore, in addition to characterizing the gross morphological differences between the transgenic and WT animals, we also sought to identify the sensitivity of *in vivo* and *ex vivo* MRI to the more subtle structural changes occurring in a subset of doxycycline-treated rTg4510 mice, relative to untreated rTg4510 mice. For morphological characterization, we employed an automated TBM pipeline for high throughput analysis of neuroanatomy in the mouse brain (Powell et al., 2016). TBM has previously been applied to a number of clinical disorders, including epilepsy (Keller and Roberts, 2008) and Alzheimer's disease (Fox et al., 1996, 2001; Hua et al., 2008). Its advantages include the ability to sensitively highlight structural differences between groups without specifying a region of interest (ROI). The technique is increasingly employed in high throughput mouse structural phenotyping studies (Lau et al., 2008; Lerch et al., 2008b; Zhang et al., 2010).

In this work, we sought to directly compare parameters which must be considered when phenotyping transgenic mice using either *in vivo* or *ex vivo* MRI. We investigated differences in image quality; the effect of formalin-fixation on post-mortem tissue structure; distortions due to the *ex vivo* tissue preparation protocol; the sensitivity of TBM to morphological changes occurring in the rTg4510 mice, scanned both *in vivo* and *ex vivo*; and the relative effect sizes detected by each paradigm.

## Materials and methods

### Transgenic animals

Generation of homozygous rTg4510 transgenic mice has been reported previously (SantaCruz et al., 2005). rTg4510 mice were licensed from the Mayo Clinic (Jacksonville Florida, USA) and bred for Eli Lilly by Taconic (Germantown, USA). Mice were imported to the UK for imaging at the Centre for Advanced Biomedical Imaging, UCL, London. All studies were carried out in accordance with the United Kingdom's Animals (Scientific Procedures) Act of 1986 and approved by the UCL internal ethics committee.

In this study, 8 female WT and 17 female rTg4510 litter-matched control mice were imaged both *in vivo* and *ex vivo* at 7.5 months of age. Of the 17 rTg4510 animals, 7 were fed with doxycycline mixed chow from 4.5 months to suppress the overexpression of tau.

### Magnetic resonance imaging

All imaging was performed using a 9.4T VNMRS horizontal bore scanner (Agilent Inc.). The key differences between the *in vivo* and *ex vivo* protocols are outlined in Table 1. For *in vivo* imaging, mice were anesthetized under 2% isoflurane and positioned in an MRI compatible head holder to minimize motion artifacts. Anesthesia was then maintained at 1.5% isoflurane in 100% O_2_ throughout imaging. Core temperature and respiration were monitored using a rectal probe and pressure pad respectively (SA instruments). Mice were maintained at ~37°C using heated water tubing and a warm air blower with a feedback system (SA Instruments).

**Table 1:** **Summary of the key differences between *in vivo* and *ex vivo* scanning protocols**.

	***In vivo***	***Ex vivo***
Sequence	Fast spin echo	Gradient echo
Weighting	T2-weighted	T2^*^-weighted
Resolution	150 μm^3^	40 μm^3^
Imaging time	1 h 30 min	11 h 24 min
Imaging coil	4 channel surface coil	Volume coil
No. of subjects imaged per acquisition	1	3
Averages	1	6
Contrast agent?	N	Y

### *In vivo* structural imaging

A 120 mm diameter imaging gradient set (SGRAD 205/120/HD/S, Agilent Technologies UK Ltd., Berkshire, UK) was used. A 72 mm birdcage radiofrequency (RF) coil was employed for RF transmission and a quadrature mouse brain surface coil (RAPID, Germany) was used for signal detection. Tuning and matching of the coil was performed manually.

A T2-weighted, three dimensional (3D) fast spin echo (FSE) sequence was implemented for structural imaging with the following parameters: Field-of-view (FOV) = 19.2 × 16.8 × 12.0 mm; resolution = 150 × 150 × 150 μm; repetition time (TR) = 2,500 ms, effective echo time (TE_eff_) = 43 ms, ETL = 4; NSA = 1. Total imaging time was ~1 h and 30 min.

### Perfusion fixation

Following *in vivo* imaging, animals were terminally anesthetized with an overdose of Euthanal administered via intraperitoneal injection. The thoracic cavities were opened and the animals were intracardially perfused through the left ventricle of the heart: first with 15–20 mL of saline (0.9%) and heparin; second with 50 mL of Formalin and 8 mM Magnevist, at a flow rate of 3 mL per minute. Following perfusion, the animal was decapitated, defleshed, and the lower jaw removed. All brains were stored in-skull at 4°C and soaked in Formalin doped with 8 mM Magnevist® for 9 weeks prior to *ex vivo* scanning (Cleary et al., 2011b).

### *Ex vivo* structural imaging

An imaging gradient set with a 60 mm inner diameter (SGRAD 115/60/HD/S, Agilent Technologies UK Ltd., Berkshire, UK) was used. A 35 mm birdcage RF coil was employed for RF transmission and signal detection. Tuning and matching of the coil was performed manually.

A custom-build three brain holder was used to acquire high resolution *ex vivo* images of multiple brains simultaneously. A 3D gradient echo (GE) sequence was implemented for structural imaging with the following parameters: FOV = 32 × 25 × 25 mm; resolution = 40 × 40 × 40 μm; *TR* = 17 ms; *TE* = 4.54 ms; flip angle = 51°; NSA = 6. Total imaging time was 11 h 36 min (Cleary et al., 2011b).

Following *ex vivo* scanning, all brains were dispatched for histology.

### Image processing

*In vivo* and *ex vivo* MR images were reconstructed using custom software written in MATLAB. A previously published calibration protocol (O'Callaghan et al., 2014) was used to adjust volume estimates to correct for scaling errors caused by the imaging gradients. Briefly, a 3D grid phantom was imaged in both gradient sets and CT data was used as a ground truth to generate absolute scaling factors that were then applied to the data.

### Tensor-based morphometry

TBM was performed on *in vivo* and *ex vivo* structural images using a fully-automated pipeline (Powell et al., 2016). Images were automatically oriented to a standard space (Right Antero-Superior) matching that of an atlas, corrected for intensity non-uniformity using the N4 algorithm (Tustison et al., 2010), and skull stripped using STEPS (Cardoso et al., 2013) to combine masks from several prior atlas images non-rigidly registered to the data. To mask our *in vivo* and *ex vivo* data, we used *in vivo* and *ex vivo* atlases published by Ma et al. (2005, 2008).

The total brain volume (TBV) was calculated from the resulting brain mask for each individual image. For *in vivo* and *ex vivo* groups, using the voxels within each mask, brain intensities were standardized using a piece-wise linear method described by Nyul et al. (2000). A multi-iteration group-wise registration (implemented in the open-source *NiftyReg* software package available from https://sourceforge.net/projects/niftyreg, Modat et al., 2010) was performed as follows to align equivalent local regions between subjects. First, all subjects were rigidly aligned to a randomly-chosen target member of the group. This was followed by four iterations of global affine registration (12 degrees of freedom) between subjects and the target image, using a block-matching algorithm (Modat et al., 2014) with normalized cross-correlation used as the similarity metric. After each iteration of registration, the group intensity average of all the transformed, resampled images was found in the space of the target, and was used as the target for the subsequent iteration of registration. Next, we performed 20 iterations of non-rigid registration (NRR), based upon symmetric free-form deformation (Modat et al., 2012). Control points were spaced at 4 voxel intervals and the similarity measure used was normalized mutual information. As the iterations progressed, the average image sharpened as alignment improved and approached the true average morphology of the group.

We transformed the deformation fields from this registration by taking the log of the determinant of the Jacobian matrix calculated at each voxel in the final average image space, to give that voxel's relative expansion or contraction from the final average image to each original image. These values were smoothed with a 0.2 mm FWHM Gaussian kernel to account for registration error and to render the values closer to a normal distribution. This was chosen to enhance physical differences between groups at approximately the same scale (the matched filter effect; Ashburner and Friston, 2001). We used the same FWHM for both *in vivo* and *ex vivo* data, so as to minimize this step as a source of variation between the methods: we did not expect the physical variation between groups to differ between *in vivo* and *ex vivo*. We performed mass-univariate statistics (two-tailed *t*-tests) at each voxel, fitting a General Linear model to reveal the most significant voxels contributing to global differences. The resulting statistical parametric maps were corrected for multiple tests using the False Discovery Rate (FDR, Genovese et al., 2002, *q* = 0.05).

### Deformation maps

To measure the degree of local variability between *ex vivo* and *in vivo* images, we registered each *ex vivo* brain to its corresponding *in vivo* counterpart, and calculated the mean positional distance at each voxel after resampling the resulting deformation fields into the *in vivo* brains' average space (Kovačević et al., 2005). The magnitude of the 3D vectors was calculated and the mean found over all *N* deformation fields, *i*:

$$\begin{array}{l} MPD\;\left(voxel\right)=\frac{1}{N}\overset{N}{\underset{i=1}{\sum }}\sqrt{x_{i}^{2}+y_{i}^{2}+z_{i}^{2}} \end{array}$$

where *x, y, z* are the vector components from the non-rigid registration only (excluding global rigid and affine transformations).

### Signal-to-noise and contrast-to-noise ratio calculations

SNR and CNR were calculated from WT mice (*n* = 3) using the following formulae:

$$\begin{array}{l} SNR=\;\frac{Mean\;signal\;}{Noise}\\ CNR=\frac{Mean\;signal_{cortex}-Mean\;signal_{corpus\;callosum}}{Noise} \end{array}$$

Signal was taken from the following ROIs: caudate putamen, cerebellum, corpus callosum, cortex, hippocampus, hypothalamus, olfactory bulb, midbrain and thalamus. The noise ROI was placed in a ghost-free region of background signal. Noise was defined as the standard deviation of the background signal. A 2-way ANOVA with *post-hoc* Sidak multiple comparisons was used to identify significant differences between SNR in the *in vivo* and *ex vivo* ROIs.

### Effect size and sample size calculation

Each dataset may be treated as a preliminary study from which to estimate the number of mice, *N*, (per group) required in a future *ex vivo* or *in vivo* study to show meaningful differences between the WT and untreated rTg4510 groups, with specified statistical power. We chose a conventional significance level of α = 0.05, a false negative rate β = 0.2, and initially assumed the WT group provided a reasonable estimation of local structural variability within the rTg4510 mouse population. To detect a 25% local volume difference between WT and untreated rTg4510 groups measured in *J*_*det*_ values, at each voxel, the required number of animals in each arm of the study is (Florey, 1993):

$$\begin{array}{l} N_{arm}=\frac{2\times {\left(Z_{\alpha /2}+Z_{1-\beta }\right)}^{2}\times WT_{stdev}^{2}}{{\left(0.25\right)}^{2}} \end{array}$$

where *Z*_α/2_ = 1.96 is the approximate number of standard deviations from the mean of a standard normal distribution of 1−(α/2) = 0.975; likewise, *Z*_1−β_ = 0.84 for 1−β = 0.8. *WT*_*stdev*_ is the standard deviation of the WT *J*_*det*_ values, from GWR, at a particular voxel. These vary across the brain depending upon local WT variability. *N*_*arm*_ is rounded up to the nearest integer.

As the actual difference between mean UT and WT *J*_*det*_ values is known *post-hoc*, the effect sizes (Cohen's d), both *in vivo* and *ex vivo*, may be visualized and compared at each voxel. This helps to quantify which regions are most different, between groups, regardless of statistical significance:

$$\begin{array}{l} \text{Cohen}\text{'}\text{s }d=\frac{UT_{mean}-WT_{mean}}{WT_{stdev}} \end{array}$$

We calculated *d* using a pooled standard deviation, replacing *WT*_*stdev*_ with σ_*pooled*_:

$$\begin{array}{l} \sigma_{pooled}=\sqrt{\frac{\left(WT_{stdev}^{2}\times \left(N_{WT}-1\right)+UT_{stdev}^{2}\times \left(N_{UT}-1\right)\right)}{N_{WT}+N_{UT}-2}} \end{array}$$

**Figure 5** shows equivalent slices from the *in vivo* and *ex vivo* group-wise average images, overlaid with the *d* and *N*_*arm*_ required at each voxel to detect a 25% variation in *J*_*det*_ from the WT mean, giving a realistic lower bound for future studies of the rTg4510 mouse.

### Histology and immunohistochemistry

Brain samples were processed using a Tissue TEK® VIP processor (GMI Inc, MN USA). After processing, sections were embedded in paraffin wax to allow coronal brain sections to be cut. Serial sections (6 μm) were taken using HM 200 and HM 355 (Thermo Scientific Microm, Germany) rotary microtomes.

Immunohistochemistry (IHC) was performed using a primary antibody for tau phosphorylated at serine 409 (PG-5; 1:800 from Peter Davies, Albert Einstein College of Medicine, NY, USA) and the neuronal marker NeuN (1:500 from Millipore; MAB377). Following de-paraffinisation and rehydration of the tissue sections, antigen retrieval was performed using the Lab Vision PT module system (Thermo Scientific), where sections were heated to 100°C for 20 min in citrate buffer (TA-250-PM1X; Thermo Scientific). Slides were transferred to a Lab Vision Autostainer (Thermo Scientific) where the following incubations were performed: 10 min in H_2_O_2_ (0.3%); 30 min in normal goat serum (1:20; Vector Laboratories); 60 min in primary antibody; 30 min in biotinylated goat anti-mouse IgG (1:200, BA9200; Vector Laboratories); 30 min avidin-biotin complex solution (PK-7100; Vector Laboratories); 5 min in 3,3′-diaminobenzidine (SK-4105; Vector Laboratories). Apart from the last two steps, PBS with 0.05% Tween-20 (PBS-T) was used for diluting reagents and washes between steps. Sections were then counterstained with haematoxylin before dehydration and cover-slipping.

To quantify the density of PG-5 and NeuN positive neurons, stained sections were digitized using the Scanscope AT slide scanner (Aperio) at 20 × magnification. Imagescope software (version 11.1.2.780; Aperio) was used to view the digitized tissue sections and delineate the boundaries of the cortex. PG-5 positive cells were manually counted within the delineated region and NeuN positive cells were quantified using a nuclear detection algorithm (Imagescope, version 11.1.2.780; Aperio); both were expressed as a percentage of the total area. These analyses were performed in a blinded fashion. A one-way ANOVA with *post-hoc* Tukey multiple comparison was performed to identify significant differences between the groups.

## Results

### Effect of *in vivo* and *ex vivo* scanning on SNR and CNR

In order to explore image quality, we investigated quantitative differences in SNR and CNR measurements from WT animals (Table 2). The SNR and CNR measurements are representative results from practical scan times achievable for the *in vivo* and *ex vivo* imaging protocols (Table 2). This was 1.5 h *in vivo*, which permitted the acquisition of other complementary functional scans within a feasible *in vivo* imaging time (Holmes et al., 2016), and 12 h to image 3 *ex vivo* brains simultaneously (4 h per *ex vivo* specimen), which maximized the utility of the MRI scanner by imaging overnight.

**Table 2:** **Mean (±SD) signal-to-noise ratio and contrast-to-noise ratio for *in vivo* and *ex vivo* wild-type mouse brains**.

**Region**	**SNR**		***P*-value**
	***In vivo* (*n* = 3)**	***Ex vivo* (*n* = 3)**	
Caudate putamen	14.4 ± 1.7	9.3 ± 1.1	≤0.001
Cerebellum	7.6 ± 0.9	11.4 ± 0.6	≤0.05
Corpus callosum	15.6 ± 0.6	4.2 ± 0.7	≤0.0001
Cortex	14.2 ± 1.4	10.0 ± 0.8	≤0.01
Hippocampus	19.3 ± 1.2	10.8 ± 0.8	≤0.0001
Hypothalamus	11.2 ± 1.2	11.5 ± 0.7	ns
Olfactory bulb	14.8 ± 1.9	10.9 ± 1.1	≤0.05
Midbrain	11.1 ± 1.2	6.9 ± 0.6	≤0.01
Thalamus	12.4 ± 1.5	7.8 ± 1.0	≤0.01
**CNR (gray-white matter)**	1.5	5.8	≤0.01

We observed an increase in SNR of the *in vivo* images compared to the *ex vivo* images in 7 of the 9 regions investigated: the caudate putamen (55%), corpus callosum (271%), cortex (42%), hippocampus (79%), olfactory bulb (36%), midbrain (61%), and thalamus (59%). Meanwhile, the SNR *ex vivo* was significantly greater in the cerebellum (50%). Conversely, we observed a three-fold increase in CNR between gray matter (cortex) and white matter (corpus callosum) structures in the *ex vivo* images compared to *in vivo* (*p* ≤ 0.01).

It is important to note the differences in the *in vivo* and *ex vivo* imaging protocols (detailed in Table 1) which will impact the SNR and CNR values. For instance, SNR is highly dependent on spatial resolution, and increases proportionally to the volume increase of the voxel. The larger voxel size (150 μm^3^ *in vivo* vs. 40 μm^3^ *ex vivo*) of the *in vivo* acquisition will have been key contributors to the increased SNR *in vivo*. Theoretically, if all other imaging parameters were the same, this increase in voxel size *in vivo* would result in an increase in SNR by a factor of ~ 53 when compared to *ex vivo* (Parker and Gullberg, 1990). However, this estimation does not take into account other differences between the imaging protocols: the use of a surface coil *in vivo* will have contributed to increased SNR relative to the *ex vivo* volume coil, whilst the high concentrations of contrast agent employed *ex vivo* will have shortened the T1 relaxation properties of the tissues and enabled the acquisition of high resolution 3D scans with improved SNR.

### Effect of perfuse-fixation on brain structure: comparison of *in vivo* and *ex vivo*

**Figure 1:**
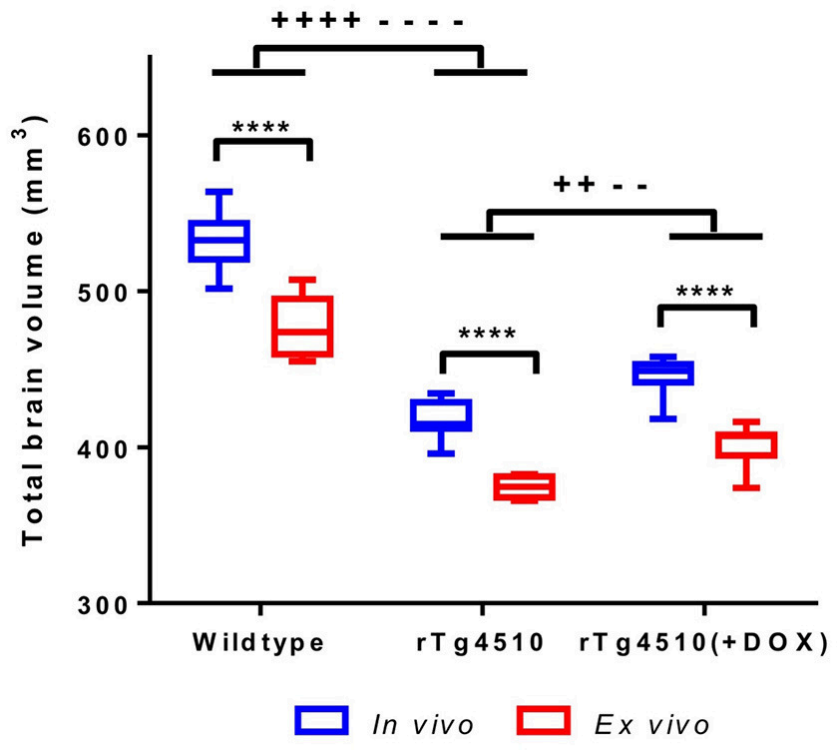
***In vivo*** **and *ex vivo* total brain volumes for wild-type, rTg4510 and rTg4510 (+DOX) animals**. *In vivo* vs. *ex vivo* comparisons: ^****^p ≤ 0.0001. *In vivo* TBV comparisons: ++*p* ≤ 0.01; ++++*p* ≤ 0.0001. *Ex vivo* TBV comparisons: −−*p* ≤ 0.01; −−−−*p* ≤ 0.0001. Whiskers represent the maximum and minimum values.

It has previously been reported that formalin fixation causes tissue shrinkage (Siegel et al., 1985; Zhang et al., 2010; Lerch et al., 2012) which may be a confounder in our *ex vivo* MRI protocol, particularly if this shrinkage is inhomogeneous across structures. In order to investigate the changes in brain morphometry occurring due to formalin fixation, we extracted total brain volumes (TBVs) for the *in vivo* and *ex vivo* mouse brain specimens using a previously published multi-atlas segmentation protocol (Ma et al., 2014). We observed a 10% reduction in *ex vivo* TBV compared to *in vivo* (*p* ≤ 0.0001) (Figure 1 and Supplementary Table 1). The degree of shrinkage was similar for WT controls (10.3%), rTg4510 mice (10.4%) and doxycycline-treated rTg4510 mice (10.1%), which suggest that the overall extent of fixation-induced shrinkage is unrelated to tau pathology in this model.

**Figure 2:**
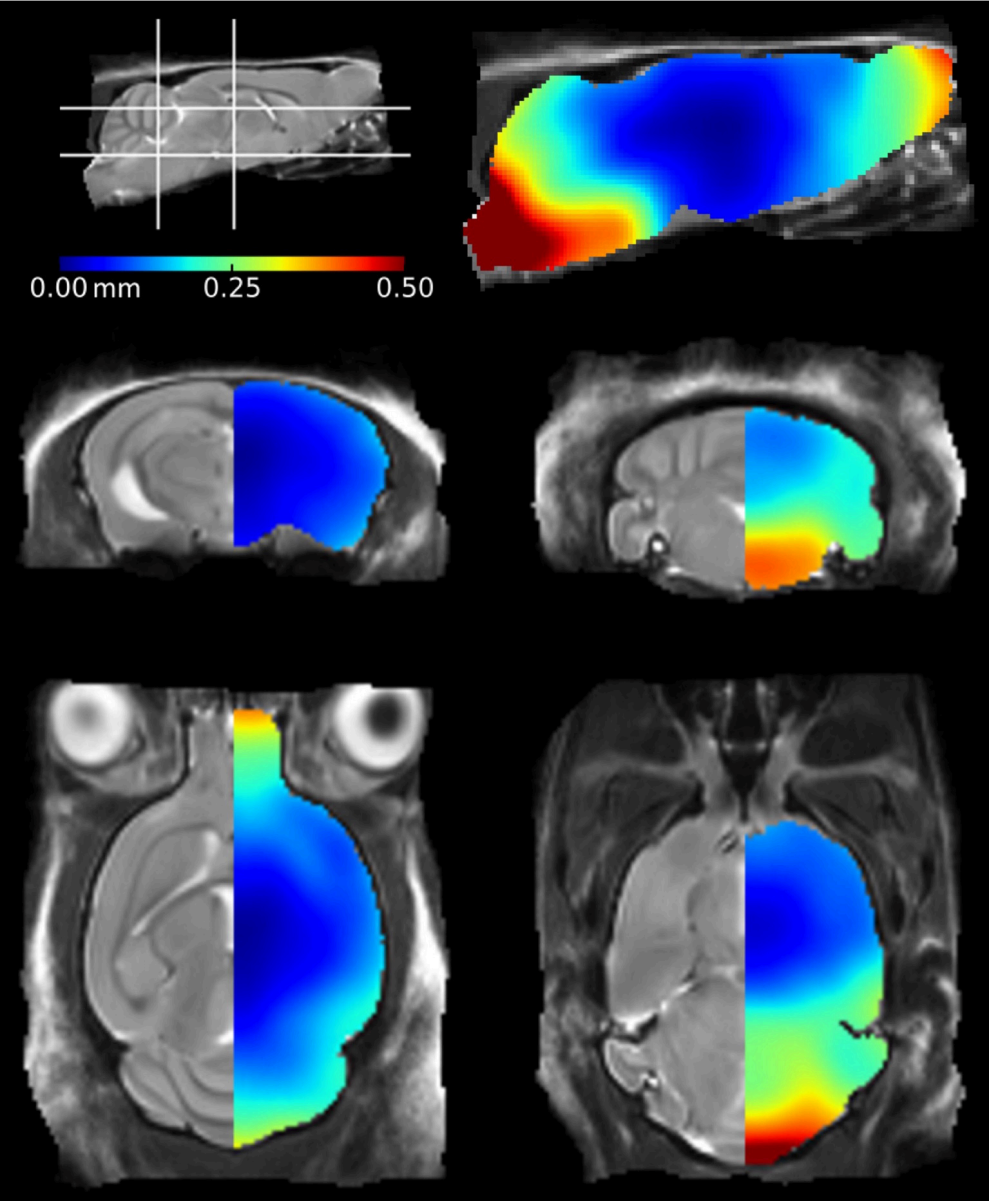
**Mean positional distance maps showing local distortions of the *ex vivo* mouse brains in comparison to their *in vivo* mouse brain template**. Deformations were calculated at a voxel-wise level. The color bar illustrates the mean distance traveled by a voxel during registration of the *ex vivo* mouse brains to the *in vivo* atlas.

We also investigated the regional effects of perfuse-fixation on brain structures by registering the *ex vivo* images to their corresponding *in vivo* mouse brain template, using methodology previously described by Ma et al. (2008). The mean positional distance map, showing mean intra-subject registration distances between *in vivo* and *ex vivo* images pairs is shown in Figure 2. The greatest changes were found within structures located frontally (the olfactory bulbs) and caudally (the brainstem). In these regions, displacements were greater than 0.25 mm can be readily visualized. The brainstem was particularly vulnerable to disturbance and exhibited displacements of up to 0.5 mm; these can be attributed to the fixation protocol which requires decapitation of the mouse. We also observed displacements within the rostral aspect of the olfactory bulbs, however the distances are markedly less than previous findings within this structure (Kovačević et al., 2005; Ma et al., 2008). The remaining brain structures, including the midbrain, hippocampus, thalamus and cortex, were relatively unaffected by perfuse-fixation. It is noticeable that the cortical surface also showed very few signs of systematic perturbation associated with the *ex vivo* protocol.

### Effect of tau pathology on brain structure: comparison of *in vivo* and *ex vivo*

We observed that the rTg4510 mice had markedly smaller brains when compared to WT (*p* ≤ 0.0001) and the doxycycline-treated rTg4510 mice (*p* ≤ 0.01) (Figure 1 and Supplementary Table 1) from both *in vivo* and *ex vivo* measurements. Importantly, the total volume differences were consistent between the *in vivo* and *ex vivo* TBV measurements: rTg4510 mice were 21% smaller that WT controls, and 16% smaller than the doxycycline-treated rTg4510 mice for both the *in vivo* and *ex vivo* cohorts.

### Tensor-based morphometry

#### Comparison between rTg4510 mice and wild-type controls

**Figure 3:**
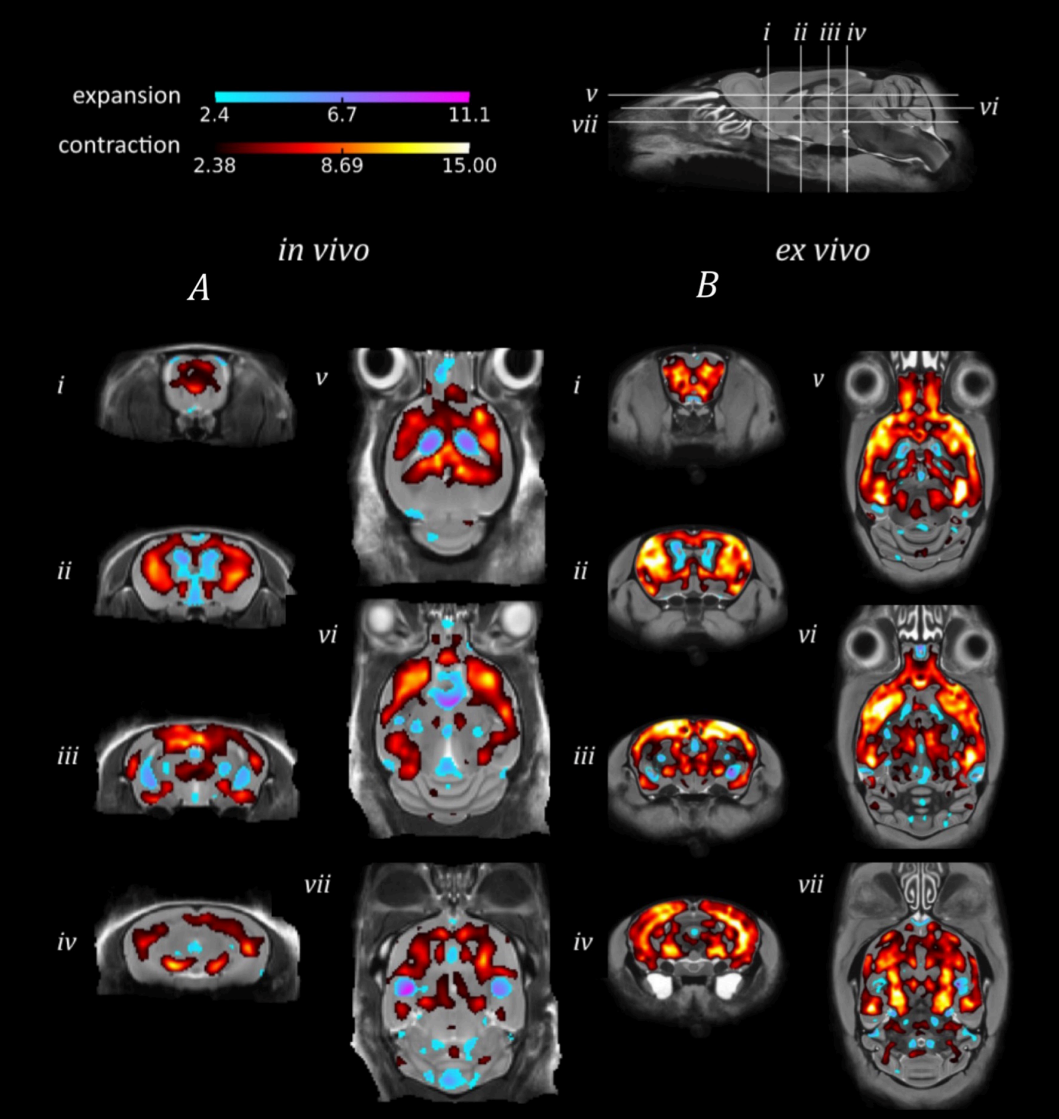
**TBM results for the morphological comparison between rTg4510 mice and wild-type controls**. Results from **(A)** *in vivo* and **(B)** *ex vivo* structural analysis, showing TBM statistical results overlaid on representative axial and coronal slices of the final group average image after 20 iterations of NRR. Red: regions where the rTg4510 brains are relatively locally smaller than the wild-type controls; blue: rTg4510 brains are locally larger. Based upon FDR-corrected t-statistics (*q* = 0.05).

The *in vivo* TBM results identified extensive bilateral atrophy within the forebrain regions, including the cortex, caudate putamen, hippocampus and hypothalamus, as well as expansion of the lateral, third and fourth ventricles (Figure 3A). Regions of expansion were also observed within the cerebellum (Figure 3Avii). A similar pattern of atrophy was also observed *ex vivo*, although these alterations appear to be more widespread in comparison to the *in vivo* findings (Figure 3B).

#### Comparison between rTg4510 mice and doxycycline-treated rTg4510 mice

**Figure 4:**
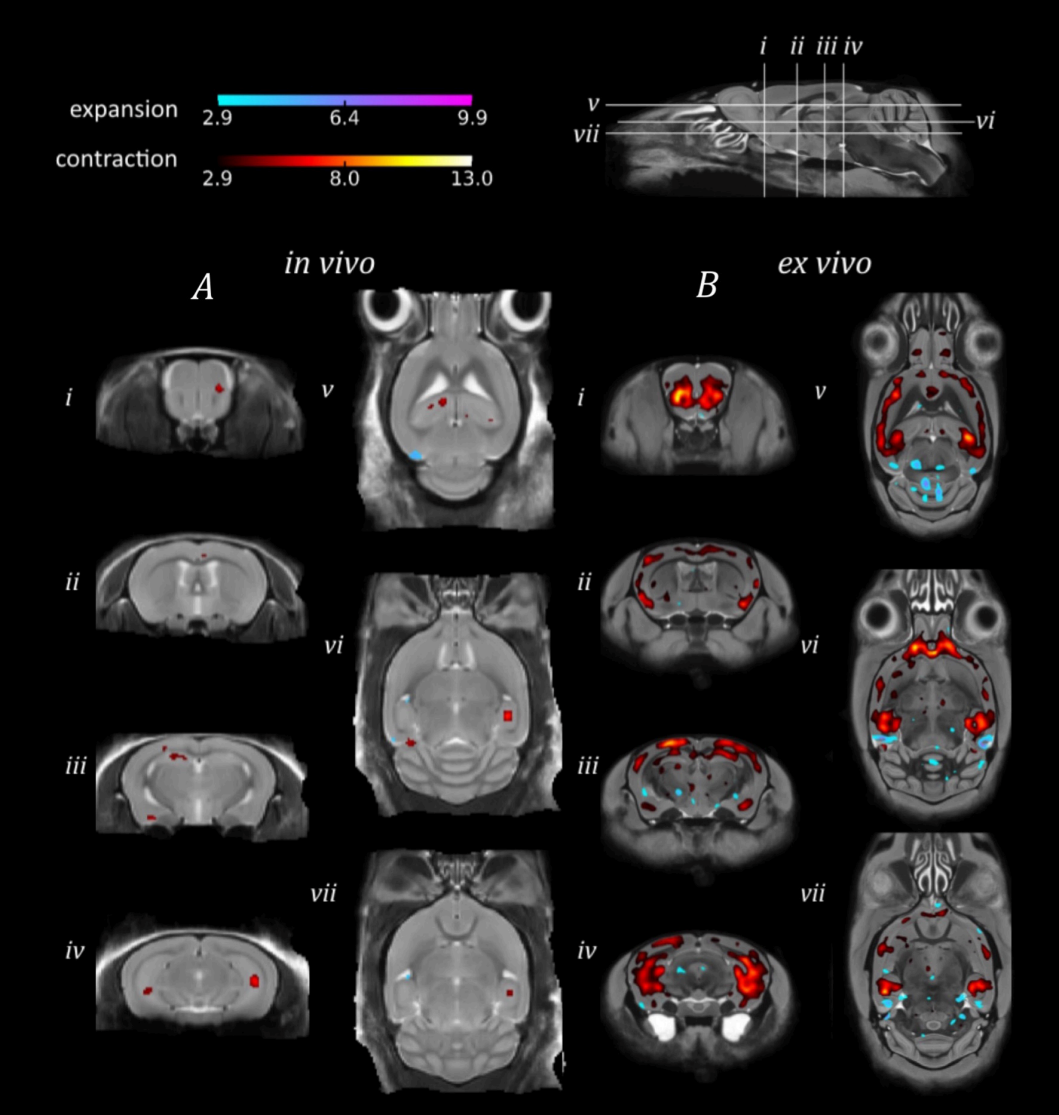
**TBM results for the morphological comparison between rTg4510 mice and doxycyline-treated rTg4510 mice**. Results from **(A)** *in vivo* and **(B)** *ex vivo* structural analysis, showing TBM statistical results overlaid on representative axial and coronal slices of the final group average image after 20 iterations of NRR. Red: regions where the rTg4510 brains are relatively locally smaller than the treated rTg4510 brains; blue: rTg4510 brains are locally larger. Based upon FDR-corrected t-statistics (*q* = 0.05).

In order to investigate the sensitivity of *in vivo* and *ex vivo* imaging to subtler structural changes, we sought to identify morphological differences between the genetically identical doxycycline-treated and untreated rTg4510 mice (Figure 4).

Due to the smaller morphological differences between the doxycycline-treated and untreated rTg4510 mice (Holmes et al., 2016), we observed correspondingly reduced regions of expansion and contraction in the *in vivo* and *ex vivo* brains (Figure 4), in contrast to the comparison of rTg4510 mice to WT controls detailed in Figure 3. The *ex vivo* results appear more sensitive in this case, and identified a relatively widespread pattern of morphometric changes, with volume loss detected in the cortex, caudate putamen and caudal hippocampal regions (Figure 4B). The *in vivo* results identified small discrete clusters of significant voxels within the piriform area of the cortex (Figure 4Aii) and caudal slices of the hippocampus (Figure 4Aiv). Although the *in vivo* findings were not as spatially extensive, we observed good regional correspondence between the TBM results observed *in vivo* and those identified *ex vivo*, with significant voxels identified in many of the same regions.

### Effect size and sample size

**Figure 5:**
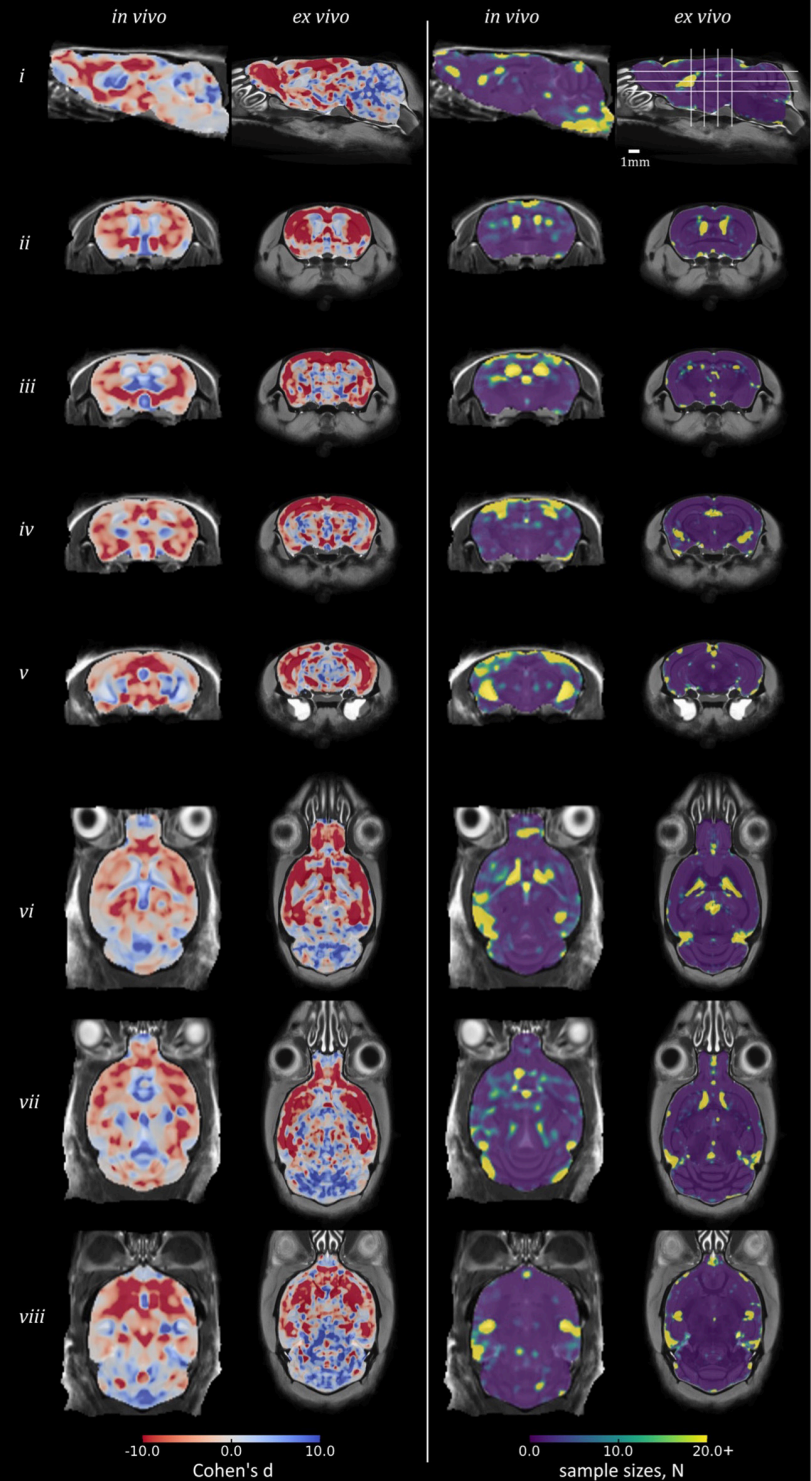
**Power analysis results in rTg4510 data compared with wild-type: Cohen's d and sample sizes**. Equivalent sagittal, coronal and transverse slices on *in vivo* and *ex vivo* GWR average images, overlaid with the Cohen's d **(left)** and sample sizes N **(right)** required to show a significant effect, with α = 0.05, β = 0.2, and an effect size 25% of the local wild-type mean volume. For Cohen's d: red, rTg4510 group locally smaller than wild-type; blue: larger.

To inform future studies in the rTg4510 mouse, we calculated Cohen's *d* and *N*_*arm*_ for the comparison of rTg4510 mice and WT controls (Figure 5). The map of Cohen's *d*, in measuring group mean separation, is similar to the thresholded statistical maps in Figure 3. The separation of groups in the hippocampus and cortex is greater *ex vivo*.

The *N*_*arm*_ analysis broadly identified that a small sample size of under 10 subjects would be sufficient to detect statistically significant differences between the WT and rTg4510 groups by performing TBM in the *ex vivo* data, across the majority of brain structures, including the midbrain and cerebellum. In order to recover structural changes occurring *in vivo*, a sample size of around 15 is required in order to ensure statistical significance of changes occurring in the cortex and forebrain regions. *N*_*arm*_ was elevated around the ventricles both *in vivo* and *ex vivo* (*N*_*arm*_ >18) although this observation was most marked *in vivo*. The *N*_*arm*_ was also elevated in the entorhinal cortex of the *in vivo* data (*N*_*arm*_ >15), suggesting that a greater sample size is required to recover changes within this structure; this is likely both due to reduced WT ventricle volumes and increased WT variance around these structures. Despite these observations, we were able to recover structural changes occurring in both of these regions *in vivo* with a reduced sample size (10 rTg4510 mice and 8 WT controls), suggesting the volume changes were greater than 25% of the WT volume.

### Immunohistochemistry: cortical PG-5 and NeuN density

In order to corroborate the MRI findings with alterations occurring at the cellular level, quantitative immunohistochemistry was performed on each of the individual WT (*n* = 8), untreated rTg4510 (*n* = 10) and treated rTg4510 (*n* = 7) mice following *ex vivo* imaging.

**Figure 6:**
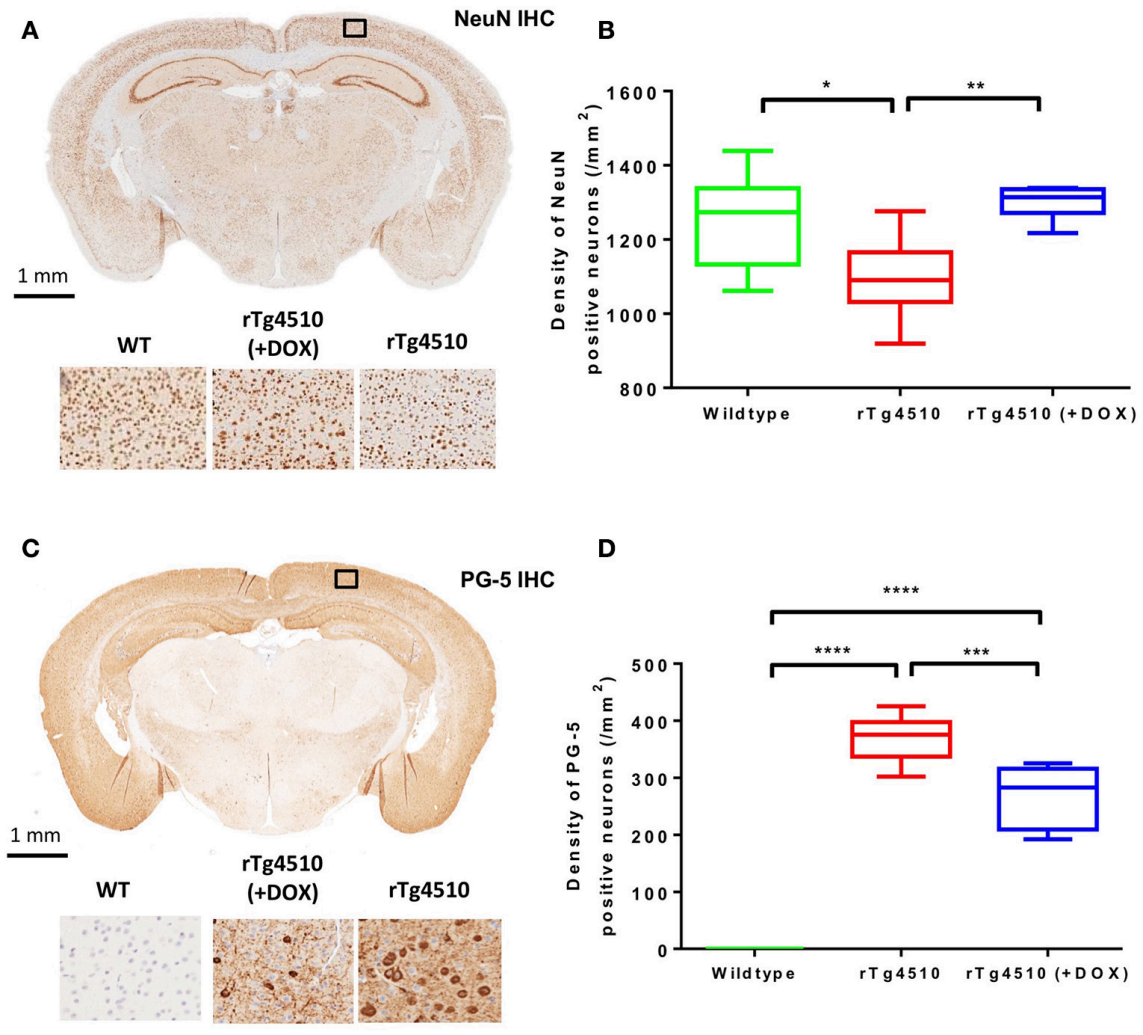
**Immunohistochemistry to estimate cortical NeuN and PG-5 positive cell density**. Representative coronal slice illustrating the distribution of **(A)** NeuN and **(C)** PG-5 positive neurons in the cortex of an untreated rTg4510 mouse. Quantitative estimates of **(B)** NeuN and **(D)** PG-5 positive cell density in the cortex for each of the 7 wild-type, 10 untreated rTg4510 and 6 treated rTg4510 mice at 7.5 months of age. Whiskers represent the maximum and minimum values. ^*^p ≤ 0.05; ^**^p ≤ 0.01; ^***^p ≤ 0.001; ^****^p ≤ 0.0001.

The distribution of NeuN positive cells can be observed in Figure 6A. The untreated rTg4510 mice showed a significant decrease in the density of NeuN positive cells in the cortex (mean NeuN density = 1,099 ± 107 cell/mm^2^) compared to WT mice (mean NeuN density = 1,253 ± 131 cell/mm^2^) (*p* ≤ 0.05) (Figure 6B). These results are consistent with previous findings, and indicate marked neurodegeneration in this model (SantaCruz et al., 2005; Wells et al., 2015). Following treatment with doxycycline, we observed a significant increase in NeuN density in the treated rTg4510 mice (mean NeuN density = 1,301 ± 44 cell/mm^2^) compared to the untreated rTg4510 mice (*p* ≤ 0.01) (Figure 6B). No significant differences were observed between NeuN cell densities in the treated rTg4510 mice compared to WT mice.

Figure 6C shows the regional distribution of PG-5 positive cells in a representative untreated rTg4510 mouse. In agreement with previous findings in this model, we observed high levels of PG-5 positive cells in the cortex of the untreated rTg4510 mice (mean PG-5 density = 369 ± 39 cell/mm^2^) (Figures 6C,D) (Wells et al., 2015; Holmes et al., 2016). No PG-5 positive cells were observed in the WT mice (Figure 6D). Following treatment with doxycycline, we observed a significant decrease in PG-5 positive cell density in the treated rTg4510 mice (mean PG-5 density = 268 ± 39 cell/mm^2^) compared to untreated rTg4510 mice (*p* ≤ 0.001) (Figure 6D).

## Discussion

With increasing numbers of rodent neuroimaging studies employing TBM to identify regions of structural change (Lerch et al., 2011a), it is important that the compromises between *in vivo* and *ex vivo* MRI are fully understood in order to maximize sensitivity to morphological differences. A number of publications have previously addressed the trade-offs associated with imaging *in vivo* and *ex vivo* mouse brains (Ma et al., 2008; Scheenstra et al., 2009; Lerch et al., 2012); in this work, we present the first application of TBM to both *in vivo* and *ex vivo* structural MRI for investigating morphological changes in a mouse model of disease: the rTg4510 model of tauopathy. In addition to characterizing the gross morphological differences in the rTg4510 mice compared to WT controls, the ability to modulate the expression of the tau transgene with doxycycline introduced subtler group differences in brain morphology to explore using *in vivo* and *ex vivo* MRI. Both were able to capture the gross atrophy occurring in the rTg4510 mice relative to WT controls, with regions highlighted by TBM aligning with post-mortem evaluation of tau pathology in this model. However, when we sought to explore the more discrete alterations in a subset of doxycycline-treated rTg4510 mice, the *ex vivo* findings were more sensitive to regions of change. A number of different factors will affect the ability of *in vivo* and *ex vivo* MRI to detect morphological changes. This discussion explores some of these in more detail, and highlights the major differences that must be considered for future neurological studies of mouse models.

Our T2-weighted *in vivo* MRI images enabled visual identification of many structures of interest in the mouse brain, including the hippocampus, caudate putamen and cortex. This was achieved in the absence of exogenous contrast agents, such as manganese, which are frequently employed in *in vivo* studies of mouse neuroanatomy (Koretsky and Silva, 2004; Yu et al., 2005). High doses of manganese are believed to produce neurotoxic effects (Thuen et al., 2008; Pan et al., 2011), which could confound longitudinal analysis by interfering with both normal healthy aging and disease progression. In this work, we instead exploited the inherent differences in T1 and T2 relaxation time to achieve adequate *in vivo* image contrast. Conversely, high resolution *ex vivo* MR images were acquired using a previously optimized protocol which employed the gadolinium-containing contrast agent Magnevist (Cleary et al., 2011b); the T1-shortening effects of gadolinium enabled the acquisition of high resolution 3D data sets within a feasible scan time (typically overnight, to maximize the efficiency of scanner usage). The protocol was adapted to permit the acquisition of three *ex vivo* mouse brains simultaneously, which improved throughput and minimized the required operator time per brain.

SNR and CNR measurements of the *in vivo* and *ex vivo* data sets allowed a quantitative assessment of image quality to support our investigation. Despite limits on scan time, the increasing availability of specialist hardware to support *in vivo* imaging studies permits the acquisition of *in vivo* data with sufficient SNR and CNR to perform informative voxel wise analyses of mouse brain morphometry. Natt et al. previously reported that SNR of 15 – 20 was sufficient to reveal anatomical details in the *in vivo* mouse brain (Natt et al., 2002); in our measurements, the majority of *in vivo* brain structures under investigation fell within these proposed boundaries.

Another important consideration when imaging *ex vivo* tissue specimens is the effect of formalin-fixation on tissue integrity. It has previously been reported that fixation of *ex vivo* brain specimens results in dehydration and subsequent reduction of the relative proton density of tissues (Shepherd et al., 2009). This can cause a reduction in SNR in *ex vivo images*, although increasing the number of signal averages helped to mitigate this effect (Liu et al., 2013). Despite the lower SNR, *ex vivo* MRI had greater gray-white matter contrast compared to the *in vivo* images; this enhancement in CNR can largely be explained by the use of the contrast agent (Kim et al., 2009). We propose that CNR may be a more important measure of image quality for TBM investigations, particularly when the similarity measure which drives the image registration algorithm depends upon contrast (we used normalized mutual information, with *NiftyReg*).

It is important to note that the differences in the *in vivo* and *ex vivo* imaging protocols will all have contributed to the differences in SNR and CNR, making it difficult to untangle and isolate an overriding factor that is influencing these values. An alternative approach for this study would have been to implement identical imaging protocols, thus eliminating the effects of hardware, sample preparation and sequence optimization on these quantitative measurements of image quality. However, given the trade-offs associated with both approaches, we believed this study was better executed by applying protocols which had been individually optimized to meet the unique demands of *in vivo* and *ex vivo* scanning.

Tissue shrinkage associated with formalin fixation is a widely accepted occurrence, yet the extent to which brain tissues suffer differential distortion is not fully known. A previous study identified 4.37% shrinkage in post-mortem WT mouse brains prepared for *ex vivo* MRI (Zhang et al., 2010), markedly less than our observation of 10.3%. This discrepancy may be due to differences in the fixation procedure, such as time spent in fixative and the use of contrast agents. The authors also did not specify whether a gradient calibration protocol was in place to estimate gradient scaling errors of the different imaging systems used for the acquisition of the *in vivo* and *ex vivo* data. An additional study reported no significant difference between *in vivo* and *ex vivo* total brain volumes in a cohort of Wistar rats (Oguz et al., 2013); however, this work was also published without specifying whether a calibration protocol was employed, and no details of the fixation procedure were provided. In both cases, if discrepancies between the respective gradient sets were not accounted for, this may cause inaccuracies in the estimation of *ex vivo* tissue shrinkage (O'Callaghan et al., 2014).

The mean positional displacement maps illustrating distortions in the *ex vivo* specimens (Figure 2) provided additional insight into the effects of the *ex vivo* preparation protocol. We identified minimum disruption to the cortical and central structures of the *ex vivo* mouse brain; these regions were of particular interest in this mouse model, as atrophy is most noticeable in the cortex and hippocampus due to the regional distribution of tau pathology (SantaCruz et al., 2005). The brain stem, meanwhile, suffered the greatest deformations as it is not protected from disturbance by the skull. A previous study identified similar disruption to the brain stem using a voxel-wise analysis (Ma et al., 2008); however, the authors reported additional disturbances in the dorsal cortical regions. These brains were removed from the skull prior to *ex vivo* imaging (Ma et al., 2005) which is likely to have caused damage to the surface structures and subsequent local deformations. The olfactory bulb, cortex and cerebellum are particularly vulnerable to damage when extracting brains from the skull (Scheenstra et al., 2009; Sawiak et al., 2012). We scanned brains in-skull, in order to preserve cortical structures and alleviate any further risks associated with the removal of brains from the skull. Despite this methodical advantage, there were still some deformations in the posterior structures, which may be due to the presence of highly variable material outside the skull, which influences the registration and which must be taken into consideration when studying rodent models with an anticipated defect within these regions.

When investigating morphological differences between the rTg4510 and WT mice, we observed a similar bilateral pattern of atrophy in the *in vivo* and *ex vivo* images using TBM. Many of the same regions were identified as suffering volume loss, including the frontal cortical regions and caudal slices of the hippocampus. These regions are selectively vulnerable to neurofibrillary tangles of tau and neuronal loss in this model (SantaCruz et al., 2005), as depicted by the immunohistochemistry results. Conversely, expansion was detected within the lateral, third and fourth ventricles. Ventricular expansion, or “ventriculomegaly” has previously been observed within AD patients in response to brain shrinkage (Apostolova et al., 2012). Interestingly, expansion of the ventricles was underestimated in the *ex vivo* images, owing to collapse of the ventricular space during formalin fixation. We also detected expansion within the cerebellum, a structure where we did not anticipate any morphological alterations. The unexpected hypertrophy may be due to compensatory neuroplastic mechanisms in response to the hippocampal volume loss, as the cerebellum has recently been identified as a key structure in spatial memory processing. Although both *in vivo* and *ex vivo* data sets revealed comparable morphological changes, our findings were more spatially extensive *ex vivo*. The power analysis results suggest that increasing the sample size may have enabled a more widespread pattern of atrophy to be detected *in vivo*.

When TBM was used to investigate subtler structural changes occurring in a cohort of doxycycline-treated rTg4510 mice, relative to untreated rTg4510 mice, the benefits of *ex vivo* MRI became more apparent. A more extensive bilateral pattern of atrophy in the untreated vs. treated rTg4510 mice was detected in the *ex vivo* data sets, which could be localized to sub-regional structures such as the CA1 hippocampal subfield. Previous work in this model has found that CA1 neuron numbers stabilize following doxycycline treatment (SantaCruz et al., 2005), a finding which may have manifested as a volume loss in the untreated rTg4510 mice compared to those treated with doxycycline. The increased CNR of the *ex vivo* data sets appeared to benefit voxel-wise tests, enabling TBM to highlight more extensive regions of difference between the groups. In addition, the higher resolution afforded by *ex vivo* MRI permits improved localization of volume changes to specific sub-regional structures. These findings suggest the increased sensitivity of *ex vivo* imaging for detecting subtle structural alterations, which may be an important consideration for future neuroimaging studies in mouse models which exhibit subtle morphological alterations.

Meanwhile, the *in vivo* TBM results for the untreated vs. treated rTg4510 mice implicated the same sub-regional structures as the *ex vivo* results, although these findings were not as spatially extensive. Nevertheless, for mouse studies with no prior knowledge regarding neurological alterations, these *in vivo* results would be sufficient to guide further histological evaluation into the underlying cellular changes underpinning the MRI findings.

The ability of TBM to detect a local volumetric expansion or contraction is dependent upon the standard deviation of *J*_*det*_ values in the groups at each voxel, *WT*_*stdev*_ (or σ_*pooled*_). This variability is dependent upon the ability of the registration algorithm to successfully align equivalent voxels, which itself depends upon constraint parameters (in *NiftyReg*, bending energy and control point spacing) as well as on the inherent image contrast of the brain structures. Low contrast regions may register poorly: structures with relatively homogeneous intensities have less information to inform registration. Here, misalignments may arise, resulting in greater local *WT*_*stdev*_, and hence a greater local effect size (or *N*_*arm*_) requirement in order to achieve the desired power. This appears to be the case in the midbrain and striatum of *the in vivo* group, where there is lower structural contrast than *ex vivo*, and consequently, greater *N*_*arm*_. Additionally, the variability in the cortex may be due to misregistrations with external material on the surface of the skull; a greater problem at lower resolutions, where the separation of this material from the brain is less distinct.

*Post-hoc* tests can inform future studies. As the *WT*_*stdev*_ and *WT*_*mean*_ vary between brain regions, the power of each test varies and different sample size estimates are prescribed, throughout the brain. Notably, around the ventricles, where structural variability between animals was higher, sample size estimates were elevated. In most brain regions, especially *ex vivo*, small group sizes appear sufficient to differentiate groups. This is also dependent upon the effect size chosen (here, 0.25 of a voxel). Smaller changes would require greater *N*_*arm*_ in each group. To obtain a useful sample size for a future study focussing on a specific brain region (e.g., the hippocampus or cortex, in the rTg4510 mouse), it would be advisable to use that region to set the *N* per study arm. The group sizes in this study were sufficient to reveal significant group differences throughout most of the brain.

## Conclusion

In this work, we explored the compromises and trade-offs between *in vivo* and *ex vivo* MRI in conjunction with TBM for detecting regions of morphometric change in the rTg4510 mouse model. Our findings support the utility of *in vivo* MRI for assessment of morphological changes in transgenic mice, where atrophy was detected in equivocal regions to *ex vivo* imaging. In addition, the *in vivo* data does not suffer from dehydration or distortion artifacts, which may confound *ex vivo* studies. Our TBV findings suggest that tissue shrinkage is similar in rTg4510 mice and WT controls, with relative preservation of key central brain structures such as the cortex and hippocampus. The TBM results also indicated that *ex vivo* MRI may offer increased sensitivity to subtle morphological changes with the same sample size, such as the response to a therapeutic intervention.

We hope that this work will help support and inform research groups working in the field of preclinical MRI, and shape their decision about the best way to image their transgenic mice.

## Author contributions

Conceived and designed the experiments: HH, NP, DM, IH, JW, NC, JO, RJ, MO, EC, SO, and ML. Performed the MRI experiments: HH, OI, IH, NC, JO, and JW. Perfuse-fixed and prepared the *ex vivo* tissues: HH and OI. Performed the histology: TM, ZA, AF, and MH. Analyzed the MRI data: NP and DM. Contributed image processing analysis tools: MC, MM, and SO. Provided the transgenic mice and contributed toward our understanding of the MRI changes: RJ, MO, and EC. Wrote, edited and critically evaluated the manuscript: HH, NP, IH, JW, EF, and ML.

### Conflict of interest statement

RJ, TM, ZA, MH, AF, MO, and EC are employees of Eli Lilly and Company. The work was carried out as part of a basic research collaboration with Eli Lilly and Company, who helped fund the imaging costs. Lilly also provided the rTg4510 mice used in this study, helped with study design and carried out histopathology in the manuscript. The other authors declare that the research was conducted in the absence of any commercial or financial relationships that could be construed as a potential conflict of interest.

## Supplementary material

Supplementary Table 1 **Total brain volumes (±SD), extracted from *in vivo* and *ex vivo* imaging data. These values are displayed box plot format in Figure 1**.

## References

[B1] Apostolova L. G. Green A. E. Babakchanian S. Hwang K. S. Chou Y. Thompson P. M. (2012). Hippocampal atrophy and ventricular enlargement in normal aging, mild cognitive impairment and Alzheimer's disease. Alzheimer Dis. Assoc. Disord. 26, 17–27. 10.1097/WAD.0b013e3182163b62 22343374 PMC3286134

[B2] Ashburner J. Friston K. J. (2001). Why voxel-based morphometry should be used. Neuroimage 14, 1238–1243. 10.1006/nimg.2001.0961 11707080

[B3] Badhwar A. Lerch J. P. Hamel E. Sled J. G. (2013). Impaired structural correlates of memory in Alzheimer's disease mice. Neuroimage 3, 290–300. 10.1016/j.nicl.2013.08.017 24273714 PMC3814975

[B4] Benveniste H. Blackband S. (2002). MR microscopy and high resolution small animal MRI: applications in neuroscience research. Prog. Neurobiol. 67, 393–420. 10.1016/S0301-0082(02)00020-5 12234501

[B5] Bock N. A. Nieman B. J. Bishop J. B. Mark Henkelman R. (2005). *In vivo* multiple-mouse MRI at 7 Tesla. Magn. Reson. Med. 54, 1311–1316. 10.1002/mrm.20683 16215960

[B6] Cahill L. S. Laliberté C. L. Ellegood J. Spring S. Gleave J. A. van Eede M. C.. (2012). Preparation of fixed mouse brains for MRI. Neuroimage 60, 933–939. 10.1016/j.neuroimage.2012.01.100 22305951

[B7] Cardoso M. J. Leung K. Modat M. Keihaninejad S. Cash D. Barnes J. (2013). STEPS: similarity and truth estimation for propagated segmentations and its application to hippocampal segmentation and brain parcelation. Med. Image Anal. 17, 671–684. 10.1016/j.media.2013.02.006 23510558

[B8] Carroll J. B. Lerch J. P. Franciosi S. Spreeuw A. Bissada N. Henkelman R. M.. (2011). Natural history of disease in the YAC128 mouse reveals a discrete signature of pathology in Huntington disease. Neurobiol. Dis. 43, 257–265. 10.1016/j.nbd.2011.03.018 21458571

[B9] Cleary J. O. Modat M. Norris F. C. Price A. N. Jayakody S. A. Martinez-Barbera J. P.. (2011a). Magnetic resonance virtual histology for embryos: 3D atlases for automated high-throughput phenotyping. Neuroimage 54, 769–778. 10.1016/j.neuroimage.2010.07.039 20656039

[B10] Cleary J. O. Wiseman F. K. Norris F. C. Price A. N. Choy M. Tybulewicz V. L. J.. (2011b). Structural correlates of active-staining following magnetic resonance microscopy in the mouse brain. Neuroimage 56, 974–983. 10.1016/j.neuroimage.2011.01.082 21310249 PMC3590453

[B11] Crawley J. N. (2008). Behavioral phenotyping strategies for mutant mice. Neuron 57, 809–818. 10.1016/j.neuron.2008.03.001 18367082

[B12] Ellegood J. Babineau B. A. Henkelman R. M. Lerch J. P. Crawley J. N. (2013). Neuroanatomical analysis of the BTBR mouse model of autism using magnetic resonance imaging and diffusion tensor imaging. Neuroimage 70, 288–300. 10.1016/j.neuroimage.2012.12.029 23275046 PMC3595420

[B13] Florey C. D. (1993). Sample size for beginners. BMJ 306, 1181–1184. 10.1136/bmj.306.6886.1181 8499826 PMC1677669

[B14] Fox N. C. Crum W. R. Scahill R. I. Stevens J. M. Janssen J. C. Rossor M. N. (2001). Imaging of onset and progression of Alzheimer's disease with voxel-compression mapping of serial magnetic resonance images. Lancet 358, 201–205. 10.1016/S0140-6736(01)05408-3 11476837

[B15] Fox N. C. Warrington E. K. Freeborough P. A. Hartikainen P. Kennedy A. M. Stevens J. M.. (1996). Presymptomatic hippocampal atrophy in Alzheimer's disease. Brain 119, 2001–2007. 10.1093/brain/119.6.2001 9010004

[B16] Gates H. Mallon A. M. Brown S. D. (2011). High-throughput mouse phenotyping. Methods 53, 394–404. 10.1016/j.ymeth.2010.12.017 21185382

[B17] Genovese C. R. Lazar N. A. Nichols T. (2002). Thresholding of statistical maps in functional neuroimaging using the false discovery rate. Neuroimage 15, 870–878. 10.1006/nimg.2001.1037 11906227

[B18] Hébert F. Grand'Maison M. Ho M. K. Lerch J. P. Hamel E. Bedell B. J. (2013). Cortical atrophy and hypoperfusion in a transgenic mouse model of Alzheimer's disease. Neurobiol. Aging 34, 1644–1652. 10.1016/j.neurobiolaging.2012.11.022 23273599

[B19] Holmes H. E. Colgan N. Ismail O. Ma D. Powell N. M. O'Callaghan J. M.. (2016). Imaging the accumulation and suppression of tau pathology using multiparametric MRI. Neurobiol. Aging 39, 184–194. 10.1016/j.neurobiolaging.2015.12.001 26923415 PMC4782737

[B20] Hua X. Leow A. D. Parikshak N. Lee S. Chiang M. C. Toga A. W.. (2008). Tensor-based morphometry as a neuroimaging biomarker for Alzheimer's disease: an MRI study of 676 AD, MCI, and normal subjects. Neuroimage 43, 458–469. 10.1016/j.neuroimage.2008.07.013 18691658 PMC3197851

[B21] Keller S. S. Roberts N. (2008). Voxel-based morphometry of temporal lobe epilepsy: an introduction and review of the literature. Epilepsia 49, 741–757. 10.1111/j.1528-1167.2007.01485.x 18177358

[B22] Kim S. Pickup S. Hsu O. Poptani H. (2009). Enhanced delineation of white matter structures of the fixed mouse brain using Gd-DTPA in microscopic MRI. NMR Biomed. 22, 303–309. 10.1002/nbm.1324 19039800

[B23] Koretsky A. P. Silva A. C. (2004). Manganese-enhanced magnetic resonance imaging (MEMRI). NMR Biomed. 17, 527–531. 10.1002/nbm.940 15617051

[B24] Kovačević N. Henderson J. T. Chan E. Lifshitz N. Bishop J. Evans A. C.. (2005). A three-dimensional MRI atlas of the mouse brain with estimates of the average and variability. Cereb. Cortex 15, 639–645. 10.1093/cercor/bhh165 15342433

[B25] Lau J. C. Lerch J. P. Sled J. G. Henkelman R. M. Evans A. C. Bedell B. J. (2008). Longitudinal neuroanatomical changes determined by deformation-based morphometry in a mouse model of Alzheimer's disease. Neuroimage 42, 19–27. 10.1016/j.neuroimage.2008.04.252 18547819

[B26] Lerch J. P. Carroll J. B. Dorr A. Spring S. Evans A. C. Hayden M. R.. (2008a). Cortical thickness measured from MRI in the YAC128 mouse model of Huntington's disease. Neuroimage 41, 243–251. 10.1016/j.neuroimage.2008.02.019 18387826

[B27] Lerch J. P. Carroll J. B. Spring S. Bertram L. N. Schwab C. Hayden M. R.. (2008b). Automated deformation analysis in the YAC128 Huntington disease mouse model. Neuroimage 39, 32–39. 10.1016/j.neuroimage.2007.08.033 17942324

[B28] Lerch J. P. Gazdzinski L. Germann J. Sled J. G. Henkelman R. M. Nieman B. J. (2012). Wanted dead or alive? The tradeoff between *in-vivo* versus *ex-vivo* MR brain imaging in the mouse. Front. Neuroinform. 6:6. 10.3389/fninf.2012.00006 22470335 PMC3311228

[B29] Lerch J. P. Sled J. G. Henkelman R. M. (2011a). MRI phenotyping of genetically altered mice. Methods Mol. Biol. 711, 349–361. 10.1007/978-1-61737-992-5_17 21279611

[B30] Lerch J. P. Yiu A. P. Martinez-Canabal A. Pekar T. Bohbot V. D. Frankland P. W.. (2011b). Maze training in mice induces MRI-detectable brain shape changes specific to the type of learning. Neuroimage 54, 2086–2095. 10.1016/j.neuroimage.2010.09.086 20932918

[B31] Liu Y. Sajja B. R. Gendelman H. E. Boska M. D. (2013). Mouse brain fixation to preserve *in vivo* manganese enhancement for *ex vivo* MEMRI. J. Magn. Reson. Imaging 38, 482–487. 10.1002/jmri.24005 23349027 PMC3638066

[B32] Ma D. Cardoso M. J. Modat M. Powell N. Wells J. Holmes H.. (2014). Automatic structural parcellation of mouse brain MRI using multi-atlas label fusion. PLoS ONE 9:e86576. 10.1371/journal.pone.0086576 24475148 PMC3903537

[B33] Ma Y. Hof P. R. Grant S. C. Blackband S. J. Bennett R. Slatest L.. (2005). A three-dimensional digital atlas database of the adult C57BL/6J mouse brain by magnetic resonance microscopy. Neuroscience 135, 1203–1215. 10.1016/j.neuroscience.2005.07.014 16165303

[B34] Ma Y. Smith D. Hof P. R. Foerster B. Hamilton S. Blackband S. J.. (2008). *In vivo* 3D digital atlas database of the adult C57BL/6J mouse brain by magnetic resonance microscopy. Front. Neuroanat. 2:1. 10.3389/neuro.05.001.2008 18958199 PMC2525925

[B35] McConville P. Moody J. B. Moffat B. A. (2005). High-throughput magnetic resonance imaging in mice for phenotyping and therapeutic evaluation. Curr. Opin. Chem. Biol. 9, 413–420. 10.1016/j.cbpa.2005.06.004 16002325

[B36] Modat M. Cardoso M. J. Daga P. Cash D. Fox N. C. Ourselin S. (2012). Inverse-consistent symmetric free form deformation, in Proceedings of Biomedical Image Registration: 5th International Workshop, WBIR, Nashville, TN, July 7-8, eds Dawant B. M. Christensen G. E. Fitzpatrick J. M. Rueckert D. (Berlin; Heidelberg: Springer), 79–88. 10.1007/978-3-642-31340-0_9

[B37] Modat M. Cash D. M. Daga P. Winston G. P. Duncan J. S. Ourselin S. (2014). Global image registration using a symmetric block-matching approach. J. Med. Imaging 1:24003. 10.1117/1.JMI.1.2.024003 26158035 PMC4478989

[B38] Modat M. Ridgway G. R. Taylor Z. A. Lehmann M. Barnes J. Hawkes D. J.. (2010). Fast free-form deformation using graphics processing units. Comput. Methods Prog. Biomed. 98, 278–284. 10.1016/j.cmpb.2009.09.002 19818524

[B39] Mouse Genome Sequencing Consortium Waterston R. H. Lindblad-Toh K. Birney E. Rogers J. Abril J. F.. (2002). Initial sequencing and comparative analysis of the mouse genome. Nature 420, 520–562. 10.1038/nature01262 12466850

[B40] Natt O. Watanabe T. Boretius S. Radulovic J. Frahm J. Michaelis T. (2002). High-resolution 3D MRI of mouse brain reveals small cerebral structures *in vivo*. J. Neurosci. Methods 120, 203–209. 10.1016/S0165-0270(02)00211-X 12385770

[B41] Norris F. C. Wong M. D. Greene N. D. E. Scambler P. J. Weaver T. Weninger W. J.. (2013). A coming of age: advanced imaging technologies for characterising the developing mouse. Trends Genet. 29, 700–711. 10.1016/j.tig.2013.08.004 24035368

[B42] Nyul L. G. Udupa J. K. Zhang X. (2000). New variants of a method of MRI scale standardization. IEEE Trans. Med. Imaging 19, 143–150. 10.1109/42.836373 10784285

[B43] O'Callaghan J. Wells J. Richardson S. Holmes H. Yu Y. Walker-Samuel S.. (2014). Is your system calibrated? MRI gradient system calibration for pre-clinical, high-resolution imaging. PLoS ONE 9:e96568. 10.1371/journal.pone.0096568 24804737 PMC4013024

[B44] Oguz I. Yaxley R. Budin F. Hoogstoel M. Lee J. Maltbie E.. (2013). Comparison of magnetic resonance imaging in live vs. post mortem rat brains. PLoS ONE 8:e71027. 10.1371/journal.pone.0071027 23967148 PMC3742751

[B45] Pan D. Caruthers S. D. Senpan A. Schmieder A. H. Wickline S. A. Lanza G. M. (2011). Revisiting an old friend: manganese-based MRI contrast agents. Wiley Interdiscip. Rev. 3, 162–173. 10.1002/wnan.116 20860051 PMC3157601

[B46] Parker D. L. Gullberg G. T. (1990). Signal-to-noise efficiency in magnetic resonance imaging. Med. Phys. 17, 250–257. 10.1118/1.596503 2333051

[B47] Powell N. M. Modat M. Cardoso M. J. Ma D. Holmes H. E. Yu Y.. (2016). Fully-automated μMRI morphometric phenotyping of the Tc1 mouse model of down syndrome. PLoS ONE 11:e0162974. 10.1371/journal.pone.0162974 27658297 PMC5033246

[B48] Qiu J. (2006). Animal research: mighty mouse. Nature 444, 814–816. 10.1038/444814a 17167453

[B49] SantaCruz K. Lewis J. Spires T. Paulson J. Kotilinek L. Ingelsson M.. (2005). Tau suppression in a neurodegenerative mouse model improves memory function. Science 309, 476–481. 10.1126/science.1113694 16020737 PMC1574647

[B50] Sawiak S. J. Wood N. I. Carpenter T. A. Morton A. J. (2012). Huntington's disease mouse models online: high-resolution MRI images with stereotaxic templates for computational neuroanatomy. PLoS ONE 7:e53361. 10.1371/journal.pone.0053361 23300918 PMC3534048

[B51] Scheenstra A. E. van de Ven R. C. van der Weerd L. van den Maagdenberg A. M. Dijkstra J. Reiber J. H. (2009). Automated segmentation of *in vivo* and *ex vivo* mouse brain magnetic resonance images. Mol. Imaging 8, 35–44. 10.2310/7290.2009.00004 19344574

[B52] Shepherd T. M. Thelwall P. E. Stanisz G. J. Blackband S. J. (2009). Aldehyde fixative solutions alter the water relaxation and diffusion properties of nervous tissue. Magn. Reson. Med. 62, 26–34. 10.1002/mrm.21977 19353660 PMC3188415

[B53] Siegel R. J. Swan K. Edwalds G. Fishbein M. C. (1985). Limitations of postmortem assessment of human coronary artery size and luminal narrowing: differential effects of tissue fixation and processing on vessels with different degrees of atherosclerosis. J. Am. Coll. Cardiol. 5, 342–346. 10.1016/S0735-1097(85)80056-5 3881498

[B54] Spring S. Lerch J. P. Wetzel M. K. Evans A. C. Henkelman R. M. (2010). Cerebral asymmetries in 12-week-old C57Bl/6J mice measured by magnetic resonance imaging. Neuroimage 50, 409–415. 10.1016/j.neuroimage.2009.12.043 20026229

[B55] Thuen M. Berry M. Pedersen T. B. Goa P. E. Summerfield M. Haraldseth O. (2008). Manganese-enhanced MRI of the rat visual pathway: acute neural toxicity, contrast enhancement, axon resolution, axonal transport, and clearance of Mn^2+^. J. Magn. Reson. Imaging 28, 855–865. 10.1002/jmri.21504 18821627

[B56] Turnbull D. H. Mori S. (2007). MRI in mouse developmental biology. NMR Biomed. 20, 265–274. 10.1002/nbm.1146 17451170 PMC2694493

[B57] Tustison N. J. Avants B. B. Cook P. A. Zheng Y. Egan A. Yushkevich P. A.. (2010). N4ITK: improved N3 bias correction. IEEE Trans. Med. Imaging 29, 1310–1320. 10.1109/TMI.2010.2046908 20378467 PMC3071855

[B58] Wells J. A. O'Callaghan J. M. Holmes H. E. Powell N. M. Johnson R. A. Siow B.. (2015). *In vivo* imaging of tau pathology using multi-parametric quantitative MRI. Neuroimage 111, 369–378. 10.1016/j.neuroimage.2015.02.023 25700953 PMC4626540

[B59] Yang D. Xie Z. Stephenson D. Morton D. Hicks C. D. Brown T. M.. (2011). Volumetric MRI and MRS provide sensitive measures of Alzheimer's disease neuropathology in inducible Tau transgenic mice (rTg4510). Neuroimage 54, 2652–2658. 10.1016/j.neuroimage.2010.10.067 21035554

[B60] Yu X. Nieman B. J. Sudarov A. Szulc K. U. Abdollahian D. J. Bhatia N.. (2011). Morphological and functional midbrain phenotypes in Fibroblast Growth Factor 17 mutant mice detected by Mn-enhanced MRI. Neuroimage 56, 1251–1258. 10.1016/j.neuroimage.2011.02.068 21356319 PMC3085550

[B61] Yu X. Wadghiri Y. Z. Sanes D. H. Turnbull D. H. (2005). *In vivo* auditory brain mapping in mice with Mn-enhanced MRI. Nat. Neurosci. 8, 961–968. 10.1038/nn1477 15924136 PMC2034206

[B62] Zhang J. Peng Q. Li Q. Jahanshad N. Hou Z. Jiang M.. (2010). Longitudinal characterization of brain atrophy of a Huntington's disease mouse model by automated morphological analyses of magnetic resonance images. Neuroimage 49, 2340–2351. 10.1016/j.neuroimage.2009.10.027 19850133 PMC2929697

