# Supplementary material for: Comparison of In Vivo and Ex Vivo MRI for the Detection of Structural Abnormalities in a Mouse Model of Tauopathy
Source: Front Neuroinform. 2017 Mar 31;11:20. doi: 10.3389/fninf.2017.00020 (PMC5374887; doi:10.3389/fninf.2017.00020)
Supplement: Supplementary Table 1 — Total brain volumes (±SD), extracted from in vivo and ex vivo imaging data. These values are displayed box plot format in Figure 1. [file Table1.DOCX]

|  | **Total brain volume (±SD) (mm^3^)** | | **Brain shrinkage (%)** |
| --- | --- | --- | --- |
|  | *In vivo* | *Ex vivo* |  |
| WT (n=8) | 532.1 (18.6) | 477.1 (19.1) | 10.3 |
| rTg4510 (n=10) | 418.5 (12.1) | 375.1 (6.5) | 10.4 |
| rTg4510(+DOX) (n=7) | 445.3 (13.0) | 400.4 (14.0) | 10.1 |
